# Food assistance use barriers, facilitators, and recommendations: insights from a qualitative study of racially and ethnically diverse parents

**DOI:** 10.1017/jns.2024.75

**Published:** 2024-11-29

**Authors:** Vivienne M. Hazzard, Alicia S. Kunin-Batson, Amanda C. Trofholz, Amy E. Noser, Junia N. de Brito, Rosabella T. Pitera, Jerica M. Berge

**Affiliations:** 1 Division of Epidemiology & Community Health, University of Minnesota School of Public Health, Minneapolis, MN, USA; 2 Department of Pediatrics, Center for Pediatric Obesity Medicine, University of Minnesota Medical School, Minneapolis, MN, USA; 3 Department of Family Medicine and Community Health, University of Minnesota Medical School, Minneapolis, MN, USA; 4 University of Minnesota Medical School, Minneapolis, MN, USA; 5 Department of Family Medicine and Adult and Child Center for Outcomes Research and Delivery Science (ACCORDS), University of Colorado Anschutz Medical Campus, Aurora, CO, USA

**Keywords:** Food assistance, Food security, Minoritised parents, Qualitative

## Abstract

The objective of this study was to explore barriers and facilitators to utilising a range of food assistance resources as reported by parents living with or at risk for food insecurity (FI), as well as parents’ recommendations for improving utilisation of these resources. Qualitative data from semi-structured interviews about parents’ perspectives on interventions to address FI were analysed using a hybrid deductive/inductive thematic approach. Parents were drawn from the larger *Family Matters* longitudinal cohort study (*N* = 1,307), which was recruited from primary care clinics in Minnesota. Forty racially and ethnically diverse parents (*M*_age_ = 38.5 years; 97.5% mothers; 85% parents of colour) were recruited by food security level, with ten parents representing each level (i.e. high, marginal, low, very low). Six overarching qualitative themes were identified, which indicated the importance of (1) comfort level seeking assistance; (2) routine screening to assess need; (3) advertising, referrals, and outreach; (4) adequacy of policies and programmes to address need; (5) resource proximity and delivery; and (6) acceptability of foods/benefits provided. With some exceptions, these themes were generally represented from more than one angle (i.e. as barriers, facilitators, recommendations) and raised as relevant across different types of assistance (e.g. federal food assistance programmes, food pantries) and different settings (e.g. schools, healthcare). This study identified key factors influencing food assistance utilisation across multiple dimensions of access. These factors—which range from psychosocial to logistical in nature—should be considered in efforts to expand the reach of food assistance programmes and, in turn, improve food security among families.

## Introduction

In recent years, over one in eight US households with children has experienced food insecurity (FI).^(1–3)^ Despite the existence of a range of food assistance resources aimed at supporting households facing FI, data show these resources are underutilised.^(1–3)^ For example, across US households that experienced FI over recent years (including households with and without children), over 40% did not utilise any of the three largest federal food assistance programmes (i.e. the Supplemental Nutrition Assistance Program (SNAP), the National School Lunch Program, and/or the Special Supplemental Nutrition Program for Women, Infants, and Children (WIC)), and only about one-third utilised food pantries.^(1–6)^ In addition to some households not being eligible to access federal food assistance programmes,^(7)^ a number of other barriers to accessing food assistance resources have been identified, including stigma, complicated application processes, and limited transportation access.^(8,9)^ Additional barriers to accessing some of these programmes are at play for certain groups, such as immigrant populations. For example, immigrants are not eligible for SNAP unless they have been lawful permanent residents for at least 5 years, are children under 18 years of age with a qualified immigration status, or meet other specific criteria.^(10)^ Additionally, fears about deportation and other potential repercussions deter participation among immigrant families in which some or all household members are eligible for SNAP.^(11)^

However, most prior studies have investigated barriers to accessing each form of food assistance in isolation. For example, barriers to accessing SNAP,^(12)^ WIC,^(13)^ the National School Lunch Program,^(14)^ and food pantries^(15)^ have each been explored in separate studies, but few studies have assessed how barriers may operate in relation to the food safety net more broadly. One such study examining barriers to using SNAP and food pantries identified a lack of transportation as a barrier to using both types of food assistance.^(16)^ As many households utilise more than one form of food assistance at a time,^(17–19)^ particularly low-income and racially and ethnically diverse households with children,^(20)^ it is crucial that our understanding of barriers to these resources—and, more importantly, our course of action to overcome these barriers—is not siloed. Moreover, with only some exceptions,^(8,14,21)^ the overwhelming majority of studies to date have focused exclusively on barriers to accessing food assistance resources, rather than balancing such a deficit-based lens with a more strengths-based lens to also explore facilitators or recommendations for improving food assistance utilisation.

To provide a more comprehensive and balanced understanding of how food assistance efforts in the United States could maximise their impact, the present study seeks to explore not only barriers but also facilitators to accessing food assistance resources broadly, as well as recommendations for improving utilisation of these resources to better ensure that families have enough to eat. To do so, this qualitative study draws upon racially and ethnically diverse parents’ lived experiences navigating the US food safety net.

## Methods

### Participants

Participants for this study were drawn from the larger *Family Matters* longitudinal cohort study (*N* = 1,307).^(22)^ Participants in the cohort study were recruited in 2016–2019 from primary care clinics in the Twin Cities area of Minnesota. Eligibility criteria included having a child between the ages of 5 and 9 years for whom they were the primary guardian and the ability to speak and read in English, Spanish, Hmong, and/or Somali. Cohort recruitment was purposely stratified to represent relatively equal numbers of African American, Hispanic/Latino, Hmong, Native American, Somali, and White households.

For the present study, qualitative one-on-one interviews were conducted by *Family Matters* study team members with parents/guardians (*n* = 40) virtually from March through May 2021. The main aim of the study was to assess how families navigate barriers and facilitators of healthy food access within their communities, as well as identify intervention targets for FI at the family, neighbourhood, school, and community levels. To represent viewpoints across the food security spectrum, recruitment was purposely stratified by recent household food security level as assessed between June 2018 and February 2021 (as part of the 18-month follow-up online survey for the larger cohort) via the six-item Short Form US Household Food Security Survey Module.^(23)^ Equal numbers of participants were recruited across the four food security levels (i.e. ten interviews each for high, marginal, low, and very low food security), with high food security households restricted to those whose incomes were below 200% of the Federal Poverty Level in order to represent households that were food secure but low income and thus at risk for FI. This approach aimed to capture perspectives of navigating the food safety net across food security levels.

Within each food security level, recruitment targets for each racial/ethnic group were set proportional to their composition in that level in the larger *Family Matters* cohort to ensure representation of racially and ethnically diverse perspectives across food security levels. Only parents/guardians who completed *Family Matters* surveys in English at baseline and 18-month follow-up were eligible for these interviews. These restrictions, along with excluding high food security households with incomes ≥200% of the Federal Poverty Level, resulted in 578 eligible families, from which randomly selected parents within strata defined by food security level and race/ethnicity were invited to participate using a staggered recruitment approach. Interested participants were directed to complete a short online form providing their availability and informed consent to participate. A total of 185 participants were sent recruitment letters to enrol the final 40 participants who ended up participating (21.6% response rate), representing 10 participants in each food security level as planned. All study procedures were approved by the University of Minnesota’s Institutional Review Board.

### Data collection

Interviews were conducted by *Family Matters* staff members who were trained in conducting qualitative interviews using a semi-structured guide.^(24)^ Interviews were conducted, recorded, and transcribed via Zoom.^(25)^ At the start of the interview, participants were given an opportunity to ask any questions regarding the consent form. They were told they could have their video camera off if they felt more comfortable. The interview guide (Supplemental Table 1) focused on four main areas: (a) general information about the participant’s home food environment, (b) the family’s experience with FI and food assistance, (c) how the COVID-19 pandemic impacted the family’s home food environment, and (d) participant suggestions for intervention targets to address FI. The average interview length was 30 min. Participants were mailed a $50 gift card after completing the virtual interview.

### Data analysis

All interview transcripts and corresponding video recordings were reviewed by *Family Matters* staff to ensure the accuracy of transcriptions. All names were changed to protect confidentiality. Cleaned and de-identified transcripts were transferred into NVivo 14 software^(26)^ and coded by two *Family Matters* team members. A hybrid deductive/inductive thematic approach was used to analyse the qualitative data.^(27)^

#### First round of coding

Prior to beginning coding, codes were entered into NVivo using the interview questions as a guide (deductive). Two coders double-coded and had consensus meetings on the first five transcripts, allowing other codes to emerge outside of prescribed interview questions (inductive). After establishing reliability, coders independently coded four transcripts (a total of eight transcripts) and then double-coded the next transcript. After double coding, a meeting was held to reach a consensus and to discuss any new codes that emerged. With this approach, 25% of interviews were double-coded. A kappa of 0.79 was achieved across all coded transcripts. The first author did not participate in the first round of coding because she had not yet been involved with this research group at the time the first round of coding was conducted.

#### Second round of coding

After the first round of coding, the first author inductively refined and organised the codes into themes with regular consultations with the larger research team. Themes were identified separately within barriers, facilitators, and recommendations. Due to substantial overlap across these domains, we present them as overarching themes.

#### Examination of relevance to type(s) of food assistance

While this study aimed to understand barriers, facilitators, and recommendations related to the use of food assistance resources broadly, the authors noticed upon completion of data analysis that some themes ended up pertaining primarily to either federal food assistance programmes (e.g. SNAP, WIC, free or reduced-price school meals) or charitable food assistance (e.g. food pantries). The relevant type(s) of food assistance are therefore noted for each theme in the presentation of results.

#### Examination of differences across household food security levels

The percentage of participants endorsing each overarching theme was compared across each household food security level to explore whether certain themes were more or less commonly endorsed at particular food security levels.

## Results

Interview participants included forty parents/primary guardians between 25 and 66 years of age. Demographic characteristics in the full sample and by household food security level are presented in Table 1. Nearly all (95%) of participants in the sample reported household participation in at least one type of federal food assistance on previous surveys from the larger *Family Matters* study (Table 2; charitable food assistance use was not assessed quantitatively in the full sample), and all parents shared personal experiences in the interviews related to using at least one type of food assistance.

**Table 1.** Demographic characteristics of the sample, overall and by recent household food security level

	Overall (*N* = 40)	Within each household food security level			
		High food security (*n* = 10)	Marginal food security (*n* = 10)	Low food security (*n* = 10)	Very low food security (*n* = 10)
	Mean or %				
Age, in years	38.5	37.8	39.2	40.4	36.5
Sex					
Female	97.5%	100%	100%	90%	100%
Male	2.5%	0%	0%	10%	0%
Race/ethnicity					
African American	37.5%	40%	30%	40%	40%
American Indian/Native American	27.5%	10%	40%	40%	20%
Hispanic/Latine	10%	0%	20%	10%	10%
Hmong	7.5%	20%	0%	0%	10%
Laotian	5%	0%	0%	10%	10%
Liberian	2.5%	0%	0%	0%	10%
Native Hawaiian/Pacific Islander	5%	0%	0%	20%	0%
Somali	5%	10%	10%	0%	0%
White	17.5%	20%	20%	20%	10%
Nativity					
Born in the United States	82.5%	80%	80%	100%	70%
Born outside the United States	17.5%	20%	20%	0%	30%

*Note*: Race and ethnicity percentages sum to >100% within some columns because some participants self-identified with >1 race or ethnicity.

**Table 2.** Previous report of household participation in federal food assistance programmes in the sample, overall and by recent household food security level

	Overall (*N* = 40)	Within each household food security level			
		High food security (*n* = 10)	Marginal food security (*n* = 10)	Low food security (*n* = 10)	Very low food security (*n* = 10)
	%				
Any of the programmes below	95%	100%	80%	100%	100%
SNAP	77.5%	90%	70%	90%	60%
WIC	40%	40%	50%	30%	40%
Free or reduced-price school meals	82.5%	90%	70%	90%	80%

*Note*: SNAP, Supplemental Nutrition Assistance Program; WIC, Special Supplemental Nutrition Program for Women, Infants, and Children. These statistics represent participants reporting household participation at the time of the baseline *Family Matters* survey in 2016–2019 and/or the 18-month follow-up *Family Matters* survey in 2018–2021. Charitable food assistance use (e.g. visiting food pantries) is not presented because it was not assessed quantitatively in the full sample.

### Qualitative themes identified

**Figure 1.**
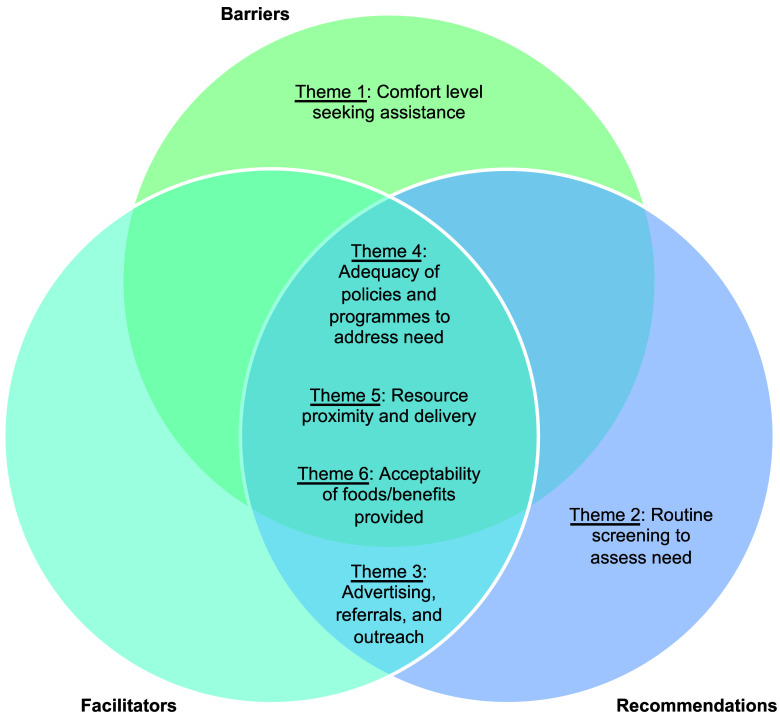
Overarching themes overlaid upon the domains of barriers, facilitators, and recommendations in which they were identified.

**Figure 2.**
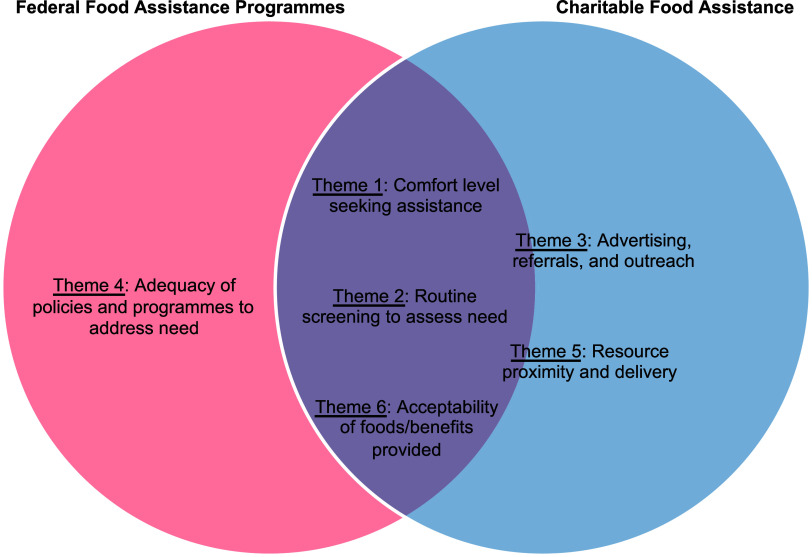
Patterning of the type(s) of food assistance identified as relevant for each overarching theme.

Across the domains of barriers, facilitators, and recommendations, six overarching qualitative themes were identified, which indicated the importance of (1) comfort level seeking assistance; (2) routine screening to assess need; (3) advertising, referrals, and outreach; (4) adequacy of policies and programmes to address need; (5) resource proximity and delivery; and (6) acceptability of foods/benefits provided. With some exceptions, these themes were generally represented across more than one domain (i.e. barriers, facilitators, recommendations), as illustrated in Fig. 1, as well as relevant across different types of food assistance, as illustrated in Fig. 2. Each theme is described in detail below with illustrative quotes.

#### Theme 1: comfort level seeking assistance

‘Not feeling comfortable’ was identified as a theme in the realm of barriers, with most quotes in this theme referring to seeking food assistance broadly, rather than referring to a specific type of assistance.

Several parents reported general discomfort seeking food assistance or discomfort centred around embarrassment, shame, or stigma:

Some of us aren’t as able to ask for help as other people. (35-year-old US-born American Indian/Native American mother, marginal food security)

People of course don’t feel comfortable saying I don’t have enough food to feed my family. That’s embarrassing. (49-year-old US-born African American and Native Hawaiian/Pacific Islander mother, low food security)

Some participants also described discomfort seeking food assistance for reasons related to prior experiences of mistreatment, as well as concerns about child protective services taking their children away:

A lot of troubling comments from [the SNAP worker] just made for a very bad experience… accusing me of lying and expressing all these things that I think were just really inappropriate for what we were there to do. (46-year-old US-born white mother, high food security)

Some people don’t reach out because they don’t want their kids taken away… when people think they can’t take care of them. (38-year-old US-born white mother, very low food security)

#### Theme 2: routine screening to assess need

Routine screening for FI, which several participants referred to as ‘checking in’, was identified as a theme in the realm of recommendations. As with the theme above, most quotes in this theme mentioned screening as relevant to seeking food assistance broadly, rather than to a specific type of assistance.

Parents proposed incorporating regular screening for FI across a variety of common settings (e.g. healthcare clinics, schools):

I think it’s important at like different points of contact to just kind of like check in and ask people if they need assistance… just because it’s like presented once as an option and someone says no doesn’t mean that it’s not worth still like bringing up or making available later on, because I think at different points of contact, there might be like different comfort levels or, you know, other circumstances might have changed where they actually feel now like ready to accept that support or that help. (35-year-old US-born white mother, marginal food security)

Just making sure that there’s communication with families, like making sure they’re getting enough food… whether there’s a doctor, whether it’s a dentist, whether it’s a school person. (41-year-old US-born Hispanic/Latina, Laotian, and white mother, low food security)

Relevant to the theme representing comfort level seeking assistance, many parents suggested screening for FI in order to identify families in need who may not otherwise seek assistance due to feelings of embarrassment and shame:

Check to make sure that you know they have enough food in their house. So that way they won’t be hungry, or you know, go unnoticed. Just check in with the families. Because a lot of people aren’t going to say they need food because they’ll probably be embarrassed. (40-year-old US-born African American mother, very low food security)

I know some people are probably ashamed. And some people don’t like to ask for help. So maybe a survey or something to see who’s in need. (35-year-old US-born African American mother, high food security)

#### Theme 3: advertising, referrals, and outreach

‘Getting the word out’ about available resources was identified as a theme across the domains of facilitators and recommendations. Quotes in this theme tended to refer to food pantries and other local food distribution sites, though some referred to connecting families with SNAP and WIC as well.

Parents reported appreciating food assistance resources that advertised their presence, both because advertising increases awareness of resource availability and because it helps to decrease stigma surrounding the use of food assistance resources:

They get the word out as best they can, to say hey, we are giving out food at this time on this day. (49-year-old US-born African American and Native Hawaiian/Pacific Islander mother, low food security)

We have like a step program food shelf that’s available, and I like that they advertise a lot or that they’re like, they’re very public with the support that they provide and so it doesn’t feel like something that’s meant to be like hidden away I guess. (35-year-old US-born white mother, marginal food security)

Parents also emphasised the usefulness of curated resource lists and having a point person available to help connect families with resources:

Connecting them with, just putting it out there, and just saying we can help you with these services, or even handing out something that has food shelf numbers to call. You know, that would help because that takes a little bit of time to go find those resources. (44-year-old US-born American Indian/Native American mother, high food security)

If you go talk to the social worker, they can get you help with more food. (42-year-old US-born Native Hawaiian/Pacific Islander mother, low food security)

Some parents highlighted the value that such a point person can offer in ways that extend beyond the simple provision of information about resources, such as by being approachable or connecting families with intermediary resources such as transportation:

Have some type of outreach where a go-to person that parents can come to and feel comfortable about communicating their needs with. (44-year-old US-born African American mother, very low food security)

Maybe if they have somebody, like some type of case manager or something that could work for people or places, or assist them with finding transportation or something. (35-year-old US-born African American mother, high food security)

#### Theme 4: adequacy of policies and programmes to address need

A theme representing the structuring and administration of policies and programmes was identified across the domains of barriers, facilitators, and recommendations. Most quotes in this theme referred to SNAP; some referred to WIC.

Many parents described issues related to eligibility, delays, administrative burden, and inadequacy of benefit amount as barriers to participating in programmes such as SNAP and WIC:

I’m in the in-between spot where I’m over the limit… not at the right position to be able to qualify for benefits, but really can’t afford, so I’m in a rock and a hard place basically in terms of qualifying for help. (44-year-old US-born African American mother, very low food security)

Getting them to look at [your paperwork] on time [is difficult]. Getting the benefits that you need in a timely matter so that you can feed your children and not starve [is difficult]. (36-year-old US-born American Indian/Native American mother, low food security)

I just feel like sometimes you have to do too much for too little. Like I said, for that little $16, I had to like make sure I get my paperwork in on time and all this stuff, and I’m like, it’s not even worth it, it’s just a few dollars, you know. (35-year-old US-born African American mother, high food security)

The renewals were always a lot of work… and at that point we were getting, you know, so little in SNAP benefits that I just let it go because it was like not worth all the trouble. (46-year-old US-born white mother, high food security)

Improvements with regard to these issues during the COVID-19 pandemic were also noted by a number of parents as facilitators to using these programmes:

Up until COVID, it was difficult, but since COVID, they’ve actually really been helpful in not having to report everything all the time, because that’s difficult to get all of that together and your income and all of that and, you know. So, this last year has been great. I mean, which is bad you know, but it’s been really helpful this last year where we haven’t had to worry so much. (44-year-old US-born American Indian/Native American mother, high food security)

[Using government resources for food] that’s become easier, or it’s only become easier since the pandemic though, before that it wasn’t readily available… There wasn’t enough before the pandemic. Barely, you know, just barely enough. But I don’t know how they did it… but they’ve definitely made the benefits more accessible. (42-year-old US-born African American mother, high food security)

Parents also made recommendations related to the structuring and administration of policies and programmes, such as simplifying the administrative processes for SNAP:

All of the forms and rules and stuff for SNAP… could be a lot more simplified. (38-year-old US-born white mother, high food security)

#### Theme 5: resource proximity and delivery

A theme representing resource proximity and delivery was identified across the domains of barriers, facilitators, and recommendations. Most quotes in this theme referred to food pantries and other free food distribution resources, while a couple referred to delivery services with SNAP.

Many parents reported obstacles to physically accessing resources, including distance from resources, lack of transportation, and difficulty bringing children with them, as barriers to using food assistance resources such as food pantries and other free food distribution resources:

If [the food distribution sites are] far, then I usually pass because I have little kids at home, and I don’t want to drag all of them with me. (33-year-old Hmong mother born outside the United States, very low food security)

I don’t have a car, but I need food. I have kids, but I don’t have the babysitter. (35-year-old US-born American Indian/Native American mother, marginal food security)

Having to get to the food pantry made it hard…Especially like when you have small children, you can’t get out the house. (42-year-old US-born white mother, marginal food security)

Conversely, proximity to resources was described as a facilitator, as were delivery services initiated during the COVID-19 pandemic:

For me, it’s the ones that are closer are helpful because I don’t drive. (38-year-old US-born African American and American Indian/Native American mother, marginal food security)

One thing that was a positive change is, you know, we use SNAP. A lot of the online grocery orders and deliveries weren’t covered, but a lot more of those opened up to using SNAP. So we can now, we can get food delivered because of COVID. That pushed it forward. So that was a positive thing. (38-year-old US-born white mother, high food security)

Many parents also made recommendations along the lines of proximity and delivery, including suggestions to incorporate food pantries or food shelves into everyday settings such as corner stores and schools:

Make it more accessible. Not having people have to go so far, to meet them in the middle… If people were able to have access to transportation, or their food delivered to them, I think that would help a lot of families. (35-year-old US-born American Indian/Native American mother, marginal food security)

Be able to deliver to people that don’t have transportation. (44-year-old US-born American Indian/Native American mother, high food security)

It’s always good to possibly have like a food pantry right on site… even corner stores, if they have a food pantry you know, I don’t know, and anything that’s close, accessible. (36-year-old US-born African American mother, low food security)

Maybe they could have, like kind of like how they have I guess food shelves, maybe they could have those in the schools. (37-year-old Liberian mother born outside the United States, very low food security)

#### Theme 6: acceptability of foods/benefits provided

Finally, the acceptability of the foods or benefits provided was identified as a theme across the domains of barriers, facilitators, and recommendations. Quotes in this theme referred to a broad range of types of food assistance, with different types of assistance tending to be referenced differentially across the domains of barriers, facilitators, and recommendations (as detailed below).

Usually, in reference to food pantries, food boxes distributed by schools during the pandemic, or occasionally WIC, several parents reported that the foods or benefits provided did not align with their families’ needs, preferences, or ability to prepare meals. In these cases, lack of acceptability served as a barrier, as well as contributed to concerns about wasting food for many parents:

My son is lactose [intolerant], so I think that they don’t realize that some things are not universal for all families. (44-year-old US-born African American mother, very low food security)

My kids don’t necessarily eat the food that they provide at the food shelf, and I don’t like to waste it. So instead of going, sometimes we didn’t just go to a food shelf because they didn’t give out stuff we ate. (35-year-old US-born American Indian/Native American mother, marginal food security)

In the past, we’ve also received WIC, which I did not find as helpful, as a lot of foods we didn’t eat. (38-year-old US-born white mother, high food security)

To just keep giving people like the ingredients isn’t really enough for a lot of families that you know, it’s not that helpful and then you end up having food waste because they’re not able to prepare it. (46-year-old US-born white mother, high food security)

Autonomy to choose foods their family would use served as a facilitator to using food assistance resources, with most quotes in this domain referring to SNAP and some referring to client choice food pantries:

The food stamps welfare was the most helpful because we can buy whatever we wanted, food we wanted to eat. (34-year-old US-born African American mother, marginal food security)

You could select what you were picking up instead of just getting whatever they gave you. (42-year-old US-born white mother, marginal food security)

In line with autonomy serving as a facilitator, many parents, such as the mother below, recommended that the community distribute gift cards that families could use to buy groceries:

Maybe the community could do like gift cards to Walmart to take the family shopping for other things that they know will that they will eat and it will go to good use. (25-year-old US-born American Indian/Native American mother, very low food security)

Parents also recommended the provision of recipes and/or classes to improve parents’ capacity to utilise foods that they may not have experience cooking with:

I think the food shelves, maybe according to their stock, they can come up with some recipes because, most of the shelves provide you with cans right? But many people don’t know how to make a very good dinner with cans. (42-year-old Hispanic/Latina mother born outside the United States, marginal food security)

Showing how to utilize food you get off of WIC or food stamps, fresh fruits and vegetables you get from the farmers’ market and having a class. (38-year-old US-born white mother, very low food security)

Lastly, parents recommended that food assistance resources offer more tailored options with consideration of factors such as cultural preferences and food allergies:

Most of the schools that I’ve seen, most of the districts are very, it’s just like generalized only to American foods, and you know some smaller or minorities say that they don’t like those kinds of food. So maybe customize it. You know, to other groups of people. (39-year-old Hmong mother born outside the United States, high food security)

Reach out and like with me for instance, I have kids on dietary restriction food allergies, being able to reach out and making sure that they can accommodate special diets and things like that. (38-year-old US-born white mother, very low food security)

### Differences across household food security levels

Percentages of participants endorsing each overarching theme by household food security level are presented in Table 3. The largest difference in percentages of participants endorsing a given theme across food security levels was observed for Theme 3 (advertising, referrals, and outreach). Specifically, 90% of parents in households with marginal food security indicated the importance of advertising, referrals, and outreach, compared to 50% of parents in households with very low food security. Themes 2 (routine screening to assess need), 4 (adequacy of policies and programmes to address need), and 5 (resource proximity and delivery) were also most commonly endorsed by parents in households with marginal food security. Theme 6 (acceptability of foods/benefits provided) was most commonly endorsed by parents in households with high food security, while Theme 1 (comfort level seeking assistance) was endorsed at the same rate across all food security levels.

**Table 3.** Percentages of participants endorsing each overarching theme by recent household food security level

	High food security (*n* = 10)	Marginal food security (*n* = 10)	Low food security (*n* = 10)	Very low food security (*n* = 10)
	% Endorsing theme within each household food security level			
Theme 1: Comfort level seeking assistance	30%	30%	30%	30%
Theme 2: Routine screening to assess need	30%	50%	30%	20%
Theme 3: Advertising, referrals, and outreach	80%	90%	70%	50%
Theme 4: Adequacy of policies and programmes to address need	60%	80%	50%	60%
Theme 5: Resource proximity and delivery	80%	90%	60%	70%
Theme 6: Acceptability of foods/benefits provided	90%	60%	80%	70%

*Note*: Percentages sum to >100% within columns because many participants endorsed >1 theme.

## Discussion

Drawing upon the perspectives of parents from racially and ethnically diverse backgrounds who have lived experience navigating the US food safety net, this qualitative study explored barriers, facilitators, and recommendations pertaining to food assistance utilisation across types of food assistance. Six overarching identified themes highlighted the importance of (1) comfort level seeking assistance; (2) routine screening to assess need; (3) advertising, referrals, and outreach; (4) adequacy of policies and programmes to address need; (5) resource proximity and delivery; and (6) acceptability of foods/benefits provided. Most of these themes were represented from more than one angle (i.e. as barriers, facilitators, recommendations) and raised as relevant across different types of assistance (e.g. SNAP, WIC, food pantries) and different settings (e.g. schools, healthcare). Every theme was endorsed across all food security levels, highlighting the relevance of these factors across food security levels. However, several themes (specifically, themes 2–5) were endorsed most often by parents in households with marginal food security, suggesting that these factors may be particularly important for improving food assistance utilisation in households with marginal food security. Findings from this study both support and expand prior research on food assistance utilisation.

Many of the findings from the present study support evidence previously reported in the literature. For example, our results cohere with prior evidence indicating that embarrassment/shame/stigma,^(8,9,28,29)^ concerns about being reported to child protective services,^(30)^ administrative burden,^(8,12,29)^ administrative delays,^(12,29,31)^ inadequate amount of benefits,^(29)^ limited transportation,^(9,32)^ and inadequate quality or limited types of foods offered^(9,14,15,28,33,34)^ serve as barriers to accessing food assistance, as well as with prior evidence that FI screening and referrals in healthcare settings,^(35,36)^ advertising and outreach,^(8,36)^ and simplified enrolment and reporting processes for SNAP^(8)^ serve as facilitators. Findings from the present study also corroborate and align with previously identified recommendations to increase federal spending for SNAP^(21)^/increase SNAP benefit amounts,^(37)^ lengthen the duration of the recertification period for SNAP,^(37)^ improve SNAP caseworker professionalism,^(29,37)^ increase awareness about food pantry existence through positive marketing messages that de-stigmatise use,^(28)^ and increase advertising of food assistance resources generally.^(29,30)^ Additionally, our results indicating that improvements in the adequacy of policies and programmes to address need, as well as in resource proximity and delivery, occurred with the COVID-19 pandemic align with prior qualitative^(38–41)^ and quantitative^(38,42,43)^ evidence suggesting that pandemic-related flexibilities and increases in benefit amounts led to increased food assistance programme participation and satisfaction. Therefore, our findings provide evidence to support the argument^(44)^ that such changes should be re-implemented and made permanent in the post-pandemic era.

Results of the present study also extend past research by taking a holistic account of parents’ perspectives regarding the use of food assistance broadly rather than focusing on a specific type of food assistance (e.g. SNAP, WIC, food pantries) in isolation as the majority of prior studies have done. In doing so, the present study highlights the broader context in which parents saw the potential for addressing FI, as well as the universality of many of the factors identified as important for predicting food assistance use. Except for the theme representing adequacy of policies and programmes to address need, which pertained primarily to federal food assistance programmes such as SNAP and WIC (perhaps reflecting a natural grouping of the data related to the administrative processes and set benefit amounts common across SNAP and WIC), all other themes identified in this study were relevant across food assistance types. Notably, however, the advertising, referrals, and outreach theme and the resource proximity and delivery theme were endorsed in reference to food pantries and other local food distribution sites more often than in reference to other types of food assistance, suggesting that these factors may be particularly important for utilisation of local resources. The relevance of these themes for food pantries and other local food distribution sites may indicate less awareness of local resources than federal food assistance programmes (i.e. necessitating more advertising and outreach for local resources) and suggest that for participants in this sample, food pantries and other food distribution sites may tend to be farther away and/or more difficult to travel to than the closest food retailer that accepts SNAP or WIC benefits. The opportunity for juxtaposition across types of food assistance also highlighted differences in how the acceptability of foods/benefits provided differentially impacts utilisation across types of food assistance. For example, *lack of acceptability* was identified as a barrier to using food boxes distributed by schools during the pandemic, some food pantries, and sometimes WIC. In contrast, *acceptability* was identified as a facilitator to using SNAP and client choice food pantries, highlighting the ability for a family to choose their own foods as key.

While not initially used as a guiding framework for our study, we observed that the themes identified in our study map closely to the dimensions of the access framework.^(45–47)^ This framework, initially introduced in the context of healthcare access, proposes that access is multi-faceted, with dimensions including awareness, approachability, availability, accommodation, affordability, and acceptability.^(45–47)^ Theme 3 in our study (advertising, referrals, and outreach) primarily aligns with the awareness dimension of access, and Theme 1 (comfort level seeking assistance) maps onto the approachability dimension. Theme 2 (routine screening to assess need) fits with both the awareness and approachability dimensions of access, in that participants discussed screening as the first step in connecting families in need with resources (thus tapping into awareness), and they also discussed screening to help overcome families’ reluctance to ask for assistance due to embarrassment or shame (thus tapping into approachability). Theme 4 (adequacy of policies and programmes to address need) aligns with both availability (i.e. relating to resources having sufficient capacity to meet demand in a timely manner^(45,47)^) and affordability (i.e. pertaining to client perception of how much the service is worth relative to the total cost—including client time—to access it^(45,47)^). Theme 5 (resource proximity and delivery) speaks to both the affordability and accommodation dimensions of access, as it emphasises factors such as limited transportation options and the need to watch young children as affordability-related barriers (e.g. the time and energy invested in taking children on the bus) and proposes accommodations such as providing delivery options. Lastly, Theme 6 (acceptability of foods/benefits provided) fits with the acceptability dimension of access. Clearly, the factors influencing food assistance utilisation identified in this study span multiple dimensions of access, suggesting the utility of a multi-pronged approach to improve food access utilisation. Although the dimensions of the access framework were developed for the purpose of improving healthcare access, it was recently proposed that this framework may also be applicable to addressing FI.^(48)^ Indeed, the parallels between the themes identified in our study and the dimensions of the access framework help solidify this framework as a useful framework for helping to better understand food assistance utilisation.

### Strengths and limitations

An important strength of this study is the racial and ethnic diversity of the participants. Considering that Black and Indigenous communities have faced unique injustices in the United States^(49)^ and, as a result, experience particularly high risk for FI,^(50–52)^ the strong representation of perspectives from Black and Indigenous families (65% of the participants) in this study can be used to help inform food assistance efforts for these populations moving forward. In addition, pre-stratification of the sample by recent food security level enabled us to represent a range of perspectives on navigating the food safety net across the food security spectrum and examine differences in themes across food security levels. This study also has limitations that should be considered. For example, all but one participant in this study identified as female, and all participants were recruited from the Twin Cities area of Minnesota. Therefore, findings may not be generalisable beyond mothers from the Twin Cities area. Additionally, only English-speaking participants were invited to participate in interviews; non-English-speaking families may face additional barriers to food assistance utilisation not represented in this study. Another feature of this study that could be considered a limitation in some regards is that it was conducted during the COVID-19 pandemic. Due to the timing of this study, some of the study findings may not be generalisable beyond the COVID-19 pandemic. For example, it is possible that pandemic-related concerns about leaving the house (e.g. fear of being exposed to COVID-19) could have contributed to the identification of the resource proximity and delivery theme. However, such concerns were not raised by participants; rather, the clearest contribution of the pandemic to this theme was the increase in food delivery services initiated by the pandemic. Thus, the timing of the study could also be argued to be a strength, as it offered participants the opportunity to juxtapose the changes to the food assistance landscape during the pandemic to pre-pandemic services, as well as highlights aspects of food assistance programmes that improved during the pandemic—aspects that could and should be continued or reinitiated, at least to some extent, in a post-pandemic era.

### Conclusions

Findings from this study highlight important factors across multiple dimensions of access that should be considered when aiming to improve food assistance utilisation and future interventions or policies addressing food security. These factors range from psychosocial (e.g. pertaining to comfort level seeking assistance) to logistical (e.g. location of services) in nature and have relevance across a variety of settings, including healthcare clinics and schools, and types of food assistance. Several of these factors were raised most often by parents in households with marginal food security, a group that may often fall through the cracks due to being considered ‘food secure’ rather than ‘food insecure’ when using the cut-off for the US Household Food Security Survey Module.^(53)^ Notably, routine screening to assess need was one of the themes endorsed most commonly by those with marginal household food security, yet screening may miss these households, bolstering the call to increase recognition of the need for food assistance at the level of marginal food security.^(54)^ Findings from this study suggest that if we can coordinate to (1) implement universal screening for FI at repeated intervals (ensuring that food assistance programmes are prepared to receive the resulting uptake in food assistance utilisation); (2) increase advertising, referrals, and outreach for food assistance resources; (3) increase comfort level seeking assistance by de-stigmatising use of food assistance and addressing concerns regarding potential ramifications of seeking assistance; (4) increase the benefit-cost ratio of participating in federal food assistance programmes by increasing benefit amounts and/or simplifying and streamlining the administrative processes; (5) offer resources at or near locations that are already built into families’ routines (e.g. their homes, schools, healthcare clinics); and (6) allow families to choose their own foods whenever possible, we would expand our reach of food assistance efforts and improve food security among families.

## Supporting information

Hazzard et al. supplementary material Hazzard et al. supplementary material

## Supplementary material

The supplementary material for this article can be found at https://doi.org/10.1017/jns.2024.75
