# Supplementary material for: Food assistance use barriers, facilitators, and recommendations: insights from a qualitative study of racially and ethnically diverse parents
Source: J Nutr Sci. 2024 Nov 29;13:e86. doi: 10.1017/jns.2024.75 (PMC11658953; doi:10.1017/jns.2024.75)
Supplement: Hazzard et al. supplementary material [file S2048679024000752sup001.docx]

**Supplemental Table 1.** *Family Matters* substudy interview guide

| 1. To get us started, I’d like to get to know your household and how meals work in your house. Who lives with you at the moment? 2. Could you share with me how often you eat together as a family and what kind of meals are prepared?   Now, I’d like to ask about how you and your family members view food security, or having enough food.   1. When you think of a family who doesn’t have access to affordable, nutritious foods in the United States, what does that look like? 2. Does this describe your current family/the family you live with now or has it ever described your current family? 3. *If yes:* Why would you say it describes your family? 4. *If yes, in the past:* What changed in your family situation to make you feel you have more/less access to affordable, nutritious foods? 5. *If no:* Why do you feel this doesn’t describe your current family situation?   4b. How might this describe your childhood experience?   1. *If food insecure (now or in the past, from question above):* What is the most challenging thing about not having enough food to feed your family? Are there other worries that you might have about food, such as wasting food?   —OR—  *If food secure (from question above):* What do you think would be the most challenging thing about not having enough food to feed your family?   1. *If food insecure (now or in the past):* What services or resources have helped (or currently are helping) when there is not enough food to feed your family?   —OR—  *If food secure:* Are there services or resources you use that help you feel you have enough food to feed your family?   1. Which resources were the most helpful? Least helpful? 2. Are there other people, such as your neighbors, family members, or friends who have assisted or supported your family to obtain food services or meals? 3. What is your experience in using or trying to obtain government resources for food? 4. How has Covid-19 changed your ability to have enough food for your family this past year? 5. Are there resources you are using now that you didn’t use prior to Covid-19? 6. How do you have discussions with your children about the food situation in your home? What prompts these conversations?   To continue, we would also like to learn about what resources could help when there is not enough food or how to improve some that already exist.   1. What do you think schools could do to help families who do not have enough food to feed their family? 2. What do you think healthcare settings, like where you or your children go for your doctor visits, and providers like family doctors and pediatricians can do to help families who do not have enough food to feed their families? 3. Are there things that community groups and organizations (e.g., food shelves) could be doing differently to help families obtain enough food? 4. Are there other places in the community that could be assisting families who do not have enough food to feed their families? 5. How can we prevent families from having to skip meals or go all day without eating? 6. Is there anything else you would like to share with us today? |
| --- |
